# A Comparison of Parametric and Non-Parametric Methods Applied to a Likert Scale

**DOI:** 10.3390/pharmacy5020026

**Published:** 2017-05-10

**Authors:** Constantin Mircioiu, Jeffrey Atkinson

**Affiliations:** 1 Pharmacy Faculty, University of Medicine and Pharmacy “Carol Davila” Bucharest, Dionisie Lupu 37, Bucharest 020021, Romania; constantin.mircioiu@yahoo.com; 2 Pharmacolor Consultants Nancy, 12 rue de Versigny, Villers 54600, France

**Keywords:** ranking, Likert, parametric, non-parametric, scores

## Abstract

A trenchant and passionate dispute over the use of parametric versus non-parametric methods for the analysis of Likert scale ordinal data has raged for the past eight decades. The answer is not a simple “yes” or “no” but is related to hypotheses, objectives, risks, and paradigms. In this paper, we took a pragmatic approach. We applied both types of methods to the analysis of actual Likert data on responses from different professional subgroups of European pharmacists regarding competencies for practice. Results obtained show that with “large” (>15) numbers of responses and similar (but clearly not normal) distributions from different subgroups, parametric and non-parametric analyses give in almost all cases the same significant or non-significant results for inter-subgroup comparisons. Parametric methods were more discriminant in the cases of non-similar conclusions. Considering that the largest differences in opinions occurred in the upper part of the 4-point Likert scale (ranks 3 “very important” and 4 “essential”), a “score analysis” based on this part of the data was undertaken. This transformation of the ordinal Likert data into binary scores produced a graphical representation that was visually easier to understand as differences were accentuated. In conclusion, in this case of Likert ordinal data with high response rates, restraining the analysis to non-parametric methods leads to a loss of information. The addition of parametric methods, graphical analysis, analysis of subsets, and transformation of data leads to more in-depth analyses.

## 1. Introduction

Statistical methods have the following as prime functions: (1) the design of hypotheses and of experimental procedures and the collection of data; (2) the synthetic presentation of data for easy, clear, and meaningful understanding; and (3) the analysis of quantitative data to provide valid conclusions on the phenomena observed. For these three main functions, two types of methods are usually applied: parametric and non-parametric. Parametric methods are based on a normal or Gaussian distribution, characterized by the mean and the standard deviation. The distribution of results is symmetric around the mean, with 95% of the results within two standard deviations of the mean. Nonparametric statistics are not based on such parameterized probability distributions or indeed on any assumptions about the probability distribution of the data. Parametric statistics are used with continuous, interval data that shows equality of intervals or differences. Non-parametric methods are applied to ordinal data, such as Likert scale data [1] involving the determination of “larger” or “smaller,” i.e., the ranking of data [2].

Discussion on whether parametric statistics can be used in a valid, robust fashion for the presentation and analysis of non-parametric data has been going on for decades [3,4,5,6]. Theoretical simulations using computer-generated data have suggested that the effects of the non-normality of distributions, unequal variances, unequal sample size, etc. on the robustness of parametric methods are not determinant [7], except in cases of very unusual distributions with a low number of data.

Regarding ordinal Likert data, the theoretical discussion of “parametric versus non-parametric” analysis continues [8,9]. In this paper, we will investigate this from a practical angle using real Likert data obtained in a recent study on pharmacy practitioners’ ranking of competencies required for pharmacy practice [10]. The differences and similarities amongst the different subgroups of pharmacists are discussed in detail in the latter paper. In this paper, we ask a specific question on statistical methodology: does the significance of the differences within and amongst subgroups of practitioners in the rankings of the importance of competencies for practice diverge with the type of analysis (parametric or non-parametric) used? We will use the data for community pharmacists and their comparison with those for industrial pharmacists as an example.

The history behind the choice of dataset for this article is as follows. The PHAR-QA project had as primary endpoint the estimation of the core competencies for pharmacy graduate students that are by and large accepted by all subgroups whatever the statistical method used; this is presented in the results section. The secondary end-point consisted in the differences between professional subgroups and we found clear differences between groups whatever the statistical method used. As is suggested by the significance of the interaction term, these differences amongst subgroups are largely centered on particular competencies (see results). This paper follows those already published on this PHAR-QA survey, and its primary purpose is to compare the use and conclusions of parametric and non-parametric analyses.

## 2. Experimental Section

The data analyzed were from an on-line survey involving 4 subgroups of respondents:

1. community pharmacists (CP, *n* = 183),
2. hospital pharmacists (HP, *n* = 188),
3. industrial pharmacists (IP, *n* = 93), and
4. pharmacists in other occupations (regulatory affairs, consultancy, wholesale, ..., OP, *n* = 72).

Respondents were asked to rank 50 competencies for practice on a 4-point Likert scale:

1 = Not important = Can be ignored.
2 = Quite important =Valuable but not obligatory.
3 = Very important = Obligatory (with exceptions depending upon field of pharmacy practice).
4 = Essential = Obligatory.

There was a “cannot rank” check box as well as a possibility of choosing not to rank at all (blank). The questionnaire response rate was calculated as the distribution between “cannot rank + choose not to rank” versus “rank (1 + 2 + 3 + 4).”

Analysis was carried out on the numbers of values for each of the 4 ranks for each of the 50 competencies. Data were also transformed into binary scores = obligatory/total% = (numbers of values for Ranks 3 and 4)/total number of values for ranks, as a percentage [11]. Such transformation leads to a loss of information but a gain in granularity and in understanding.

Results are presented in three sections starting with reflections on the distribution of the data. This is followed by a section of parametric and non-parametric presentation of the data and a final section on parametric and non-parametric analyses of the data. Data were analyzed using GraphPad software [12] and in-house Excel spreadsheets.

## 3. Results and Discussion

### 3.1. Distribution of the Data

The questionnaire response rate between “cannot rank + choose not to rank” versus “rank” was globally 14.5:85.5 (*n* = 536 respondents); there were no significant differences in response rate amongst the four subgroups (chi-square, *p* > 0.05). This aspect was not pursued further given that the vast majority of respondents (86%) were able to understand and reply to the 50 questions on competencies. It can be inferred that differences in distributions of ranking values were not based on misunderstanding of questions.

There were no differences amongst subgroups in the response rate for individual competencies (= number of responses/50) (chi-square, *p* > 0.05). Missing values were not replaced.

**Figure 1:**
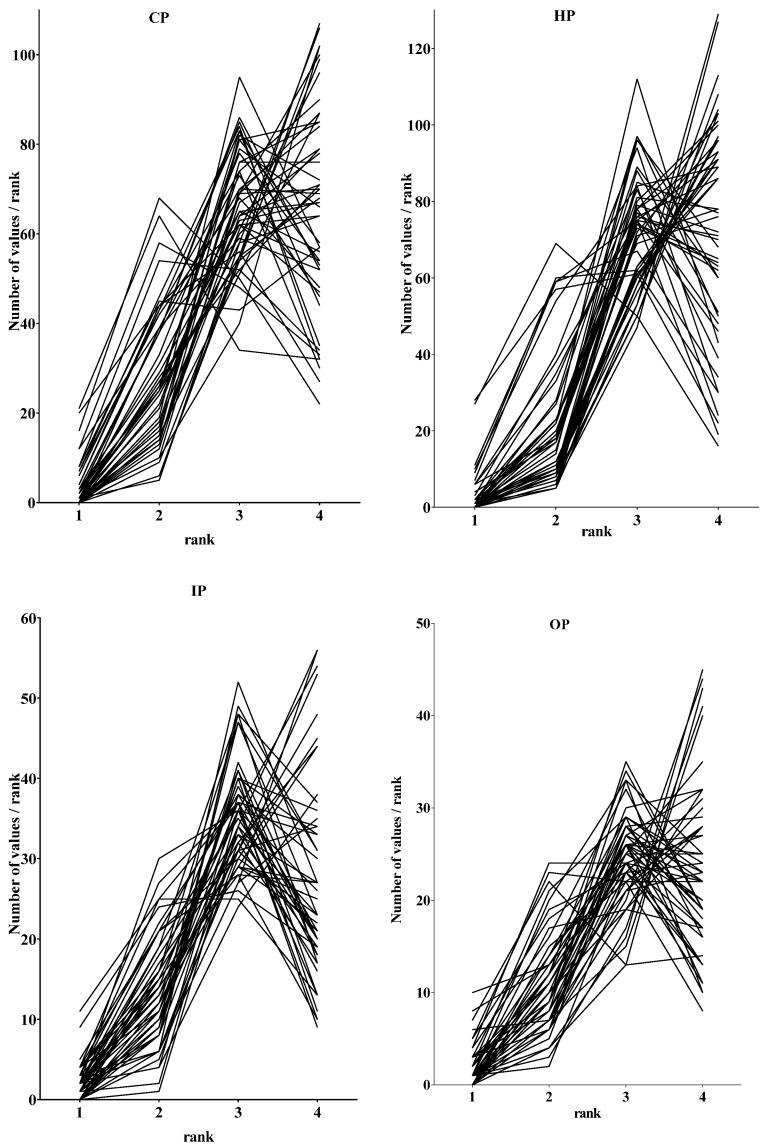
Distributions of ranking data (number of values/rank) for each of the 50 ranked competencies (lines). The four subgroups are as follows: community pharmacists (CP, *n* = 183 respondents, top left); hospital pharmacists (HP, *n* = 188, top right); industrial pharmacists (IP, *n* = 93, bottom right); pharmacists in other occupations such as regulatory affairs, consultancy, and wholesale (OP, *n* = 72, bottom left).

The distributions of the ranking data are shown in Figure 1.

Visual inspection of the four graphs reveals that there were no outliers. Distributions visually suggested a non-Gaussian distribution, i.e., neither continuous nor bell-shaped. Given the small numbers of bins involved (*n* = 4 ranks), tests of normality of distribution such as the Kolmogorov–Smirnov test were not performed.

Distributions were, however, very similar in all four subgroups. They were of two types: inverted “j” or “linear/exponential”; both types of distribution were skewed to the left, i.e., to higher ranking values (on the right of each graph). In order to estimate the numbers of each type of distribution in individual subgroups of pharmacists, the “inverted j” was defined as having a negative value for “number of values for Rank 4–number of values for Rank 3”, and the “linear/exponential” was defined as having a positive value for Rank 4–Rank 3.

The “inverted j” distribution was defined as having a negative value for “number of values for Rank 4–number of values for Rank 3”, and the “linear/exponential” distribution was defined as having a positive value for “number of values for Rank 4–number of values for Rank 3.”

Table 1 shows the numbers of “inverted j” and “linear/exponential” distributions. Chi-square analysis showed a difference between IP and the other three subgroups (*p* < 0.05). This is also seen in the visual inspection of the graphical representation in Figure 1. Distributions of negative and positive values were normal in all four subgroups; means of values “Rank 4–Rank 3” were not different from zero (*p* > 0.05).

**Figure 2:**
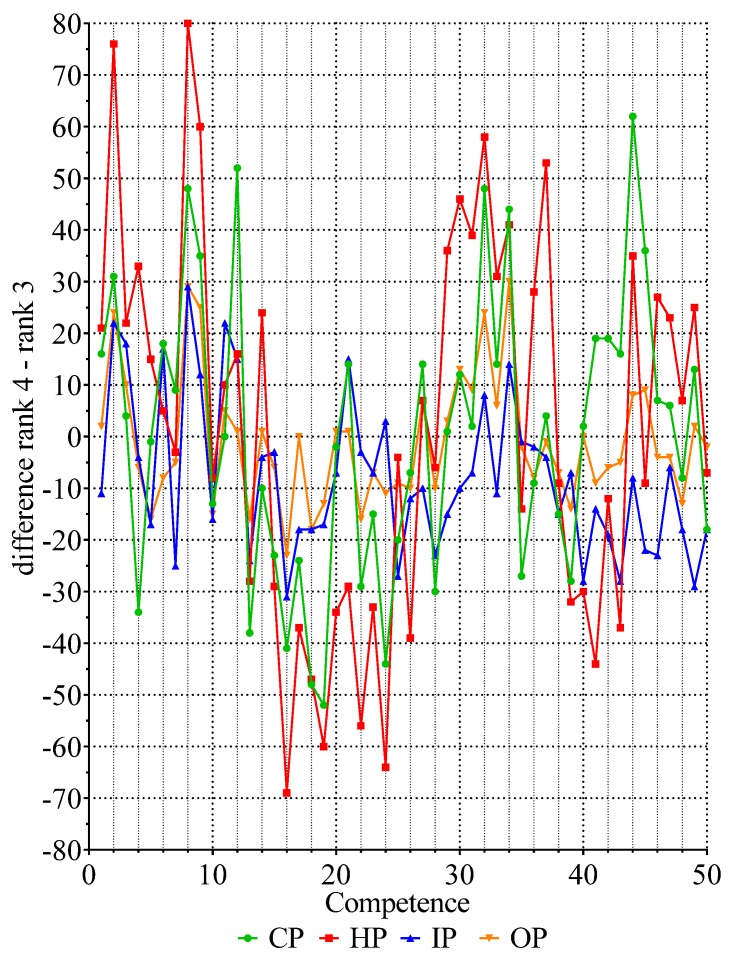
Values for the difference Rank 4–Rank 3 for all four subgroups. The four subgroups are as follows: community pharmacists (CP, *n* = 183 respondents, green circles); hospital pharmacists (HP, *n* = 188, red squares); industrial pharmacists (IP, *n* = 93, blue triangles); pharmacists in other occupations such as regulatory affairs, consultancy, and wholesale (OP, *n* = 72, orange inverted triangles).

Figure 2 contains the values for the differences in “number of values for Rank 4–number of values for Rank 3” for 50 competencies in the four subgroups. There were two clusters of negative values for competencies 13–30 and 38–50, indicating distributions of the “inverted j” form and two clusters of positive values for competencies 1–13 and 31–37, indicating “linear/exponential” distributions of ranking data. Thus, although sample distributions of ranks within competencies are not normal, they are similar in form from one competency to another, and one subgroup of pharmacists to another.

The situation here is one of similar distributions with different numbers of values (ranging from 72 for OP to 188 for HP). Boneau [7], using simulated data, found that, if numbers were large enough (>15), such a situation should not be problematic in terms of parametric analysis. Below, we shall determine whether this statement applies to the actual data.

### 3.2. Presentation and Analysis of Within-Subgroup Data

The question asked here is as follows: Within a given subgroup (CP will be used as an example), are there significant differences amongst the 50 competencies?

**Figure 3:**
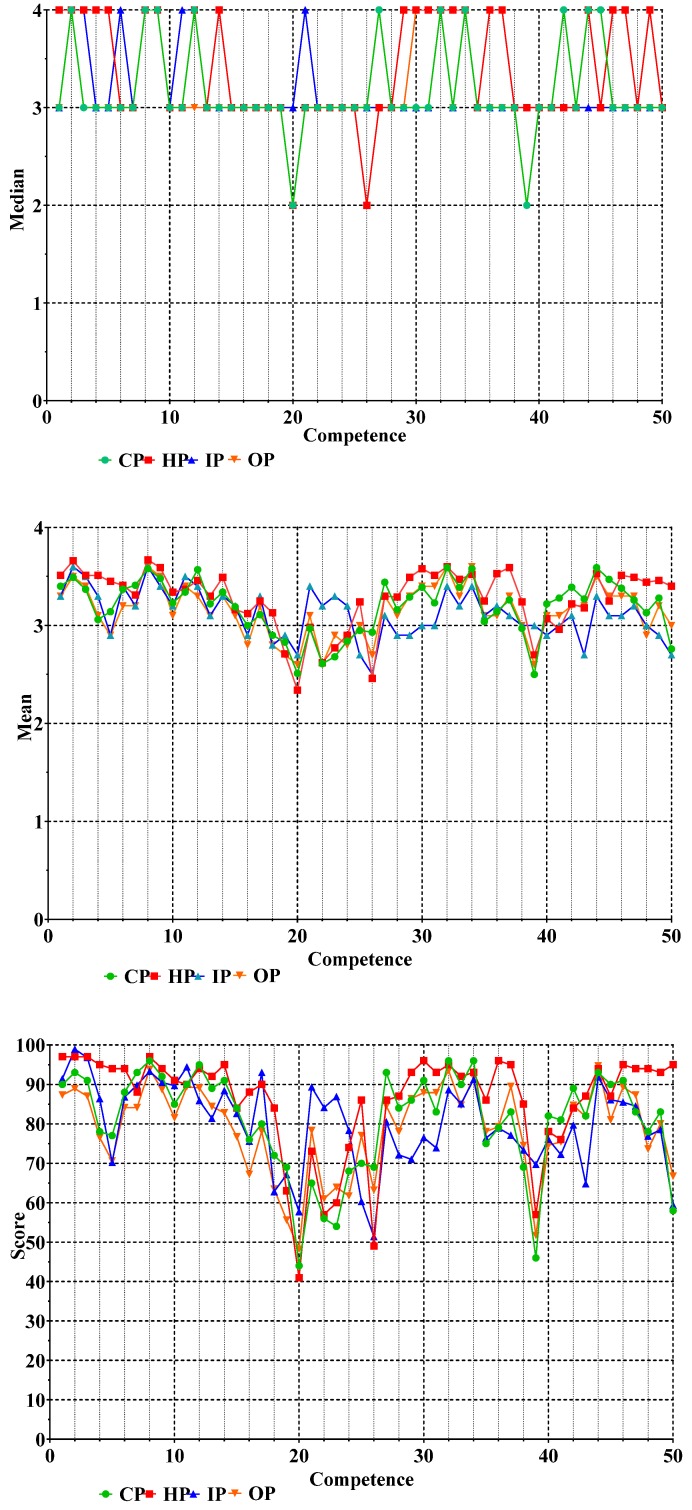
Graphic presentation of the data for the ranking of the 50 competencies. The four subgroups are as follows: community pharmacists (CP, *n* = 183 respondents, green circles), hospital pharmacists (HP, *n* = 188, red squares), industrial pharmacists (IP, *n* = 93, blue triangles), and pharmacists in other occupations such as regulatory affairs, consultancy, and wholesale (OP, *n* = 72, orange inverted triangles).

Graphic presentations of the medians, means, and scores of data for the ranking of the 50 competencies by CP, HP, IP, and OP are given in Figure 3.

For CP, whichever form of graphical presentation is used, the major features were the same, namely, that competencies 2, 8, 9, 12, 27, 32, 34, 42, 44, and 45 were ranked higher, and competencies 20 and 39 lower, than the others. The graphs for means and scores visually suggest that there may be significant differences amongst the other 38 competencies as more discriminant information is gathered by the use of parametric statistics (means) and data transformation (scores).

**Figure 4:**
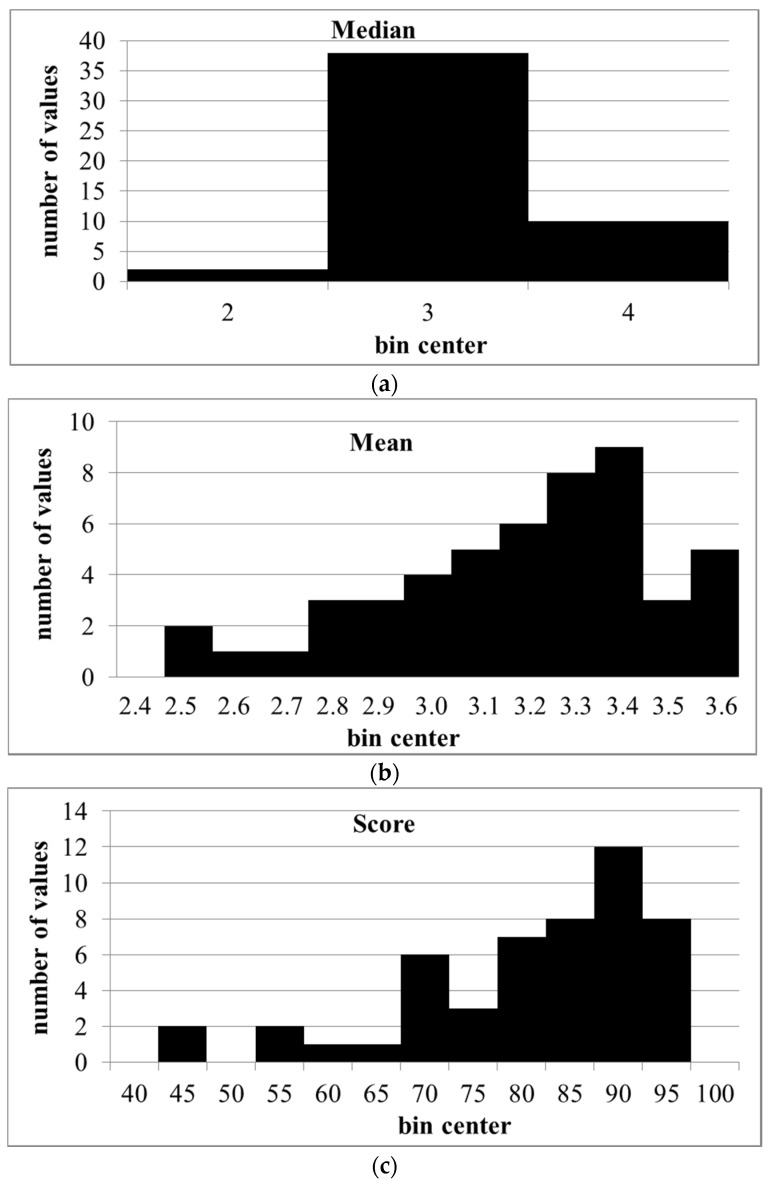
Distributions of medians, means, and scores of ranks for competencies given by CP (same data as in Figure 3). (**a**): medians; (**b**): means; (**c**): scores.

Although somewhat skewed to the right, the distributions of the means and scores were not significantly different from normal (Shapiro–Wilk and Kolmogorov–Smirnov test, *p* < 0.05). The number of bins was too small to test the distribution of medians (Figure 4).

To test for significant differences amongst rankings for comparisons between competencies across subgroups, we used (1) parametric 1-way ANOVA followed by the Bonferroni multiple comparisons test and (2) non-parametric Kruskal–Wallis analysis followed by the Dunn multiple comparisons test. Both analyses showed that there was a significant effect of “competency” (Table 2); both analyses gave the same very low *p*-values.

There were 8095 data points analyzed with 1055 missing values (11.5% of total (= 50 × 183 = 9150)). Missing values were not replaced.

The total number of possible multiple comparisons amongst the 50 competencies was 1225. There was agreement between the parametric and non-parametric tests in the case of a conclusion of “not significant” (756 cases) (Table 3). The Bonferroni test revealed a significant difference in 469/1225 = 38% of the comparisons. There was disagreement between the parametric Bonferroni test and the non-parametric Dunn test in 76 (6%) of these cases, the Bonferroni producing a significant result but not the Dunn test (Table 3).

The similarity of difference of competency-ranking (Table 3) by parametric and non-parametric methods can be formally assessed by the kappa test [13].

In this case, *P_o_* = (proportion of observed agreement) = 0.94 and *P_r_* = (proportion of random agreement) = 0.54.

$$\kappa =\frac{p_{o}-p_{r}}{1-p_{r}}=0.86$$

As we obtained a value 0.86, this can be considered as very good agreement.

In summary, both tests revealed significant and non-significant differences. In the majority of cases, the tests indicated the same result. The parametric Bonferroni test detected more significant differences than the non-parametric Dunn test, showing that the parametric test was more discriminate.

### 3.3. Presentation and Analysis of Amongst-Subgroup Data

The question asked was as follows: Are there significant differences between subgroups for one or several of the 50 competencies?

Figure 3 (above) shows the ranking data for the four subgroups in the form of medians (upper), means (middle), and scores (lower). Differences amongst subgroups are difficult to see in the case of medians. Means reveal granularity in results for the different subgroups. This shows, for example, that results for competencies 21–23 and 28–30 as ranked by IP (triangles) appear different from those of the other subgroups such as CP (circles). Such differences are accentuated in the graph of scores.

Individual ranking data for each competency in each subgroup were analyzed using a parametric two-way ANOVA with Sidak’s multiple comparisons test, and the non-parametric Friedman test with Dunn’s multiple comparisons test analyses (Table 4), in order to determine differences amongst subgroups.

The parametric two-way ANOVA revealed a significant effect of competency, subgroup, and the interaction “subgroup–competency” (Table 4). The percentage variation for competency was much greater than that for subgroup, suggesting that global differences amongst competencies were much greater than those amongst subgroups. Sidak’s multiple comparisons test (Table 4) showed a significant difference between CP and IP or OP. Although the interaction “subgroup–competency” is highly significant, this type of analysis does not permit any conclusion as to which specific competencies are significantly different between two given subgroups (this will be dealt with later using the parametric multiple *t*-test and the non-parametric chi-square test). It could be argued that the interaction effect (F-value = 3.6) could be a spurious consequence of the relatively large primary competency effect (F-value = 38). We consider that the interaction effect is not spurious. The interaction effect is real since there are special clusters of competencies that are ranked differently in different professional subgroups (see Figure 3, e.g., CP versus IP for competencies 21–23).

The large number of missing values in this two-way ANOVA (38% of total) emphasizes the unbalanced nature of the analysis with numbers per subgroup ranging from 188 (HP) to 72 (OP). This can often occur in real-life surveys.

Non-parametric Friedman analysis (Table 4) also revealed a significant overall effect of subgroup, but Dunn’s multiple comparisons test failed to reveal any significant effect of any specific combination of subgroup. It was thus less discriminant than Sidak’s parametric multiple comparisons test. Furthermore, the Friedman test does not allow for the evaluation of the significance of interactions and so again provides less information than the two-way ANOVA.

Differences in specific competencies between two given subgroups were analyzed using the parametric multiple *t*-test and the non-parametric chi-square test. Amongst the multitude of potential combinations, data are shown (Table 5) for the comparisons between CP and IP for the six competencies revealed in Figure 3 above.

**Figure 5:**
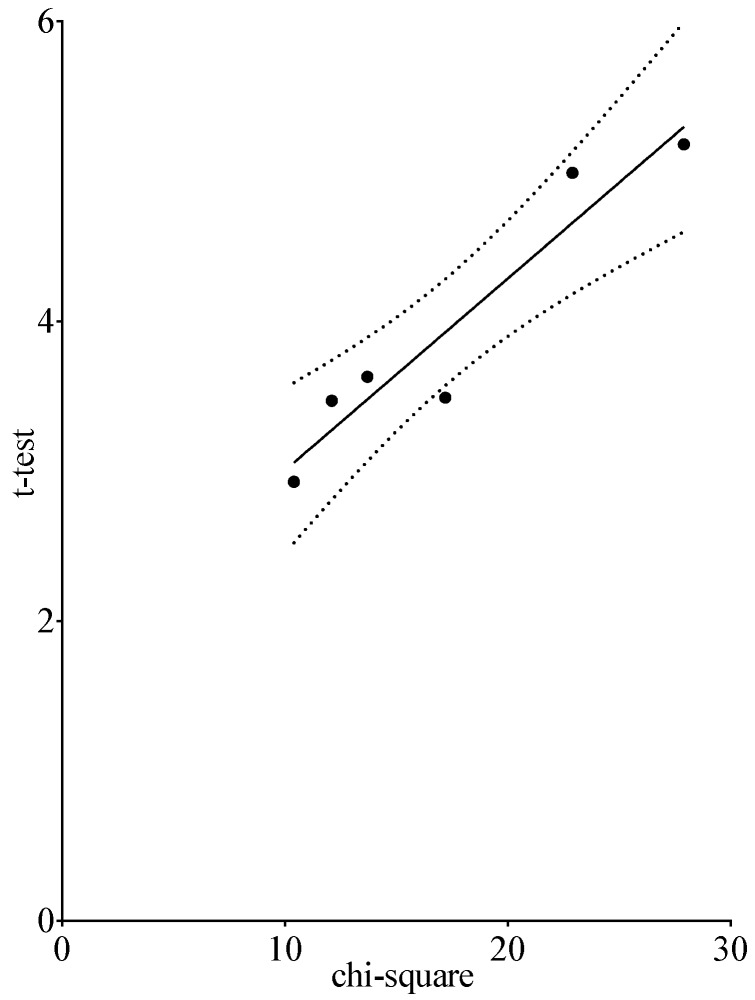
Correlation between the chi-square test and *t*-test for the competencies given in Table 5 in the comparison CP versus IP. (*t*-test = ((0.13 × chi-square) + 1.73), r^2^ = 0.91).

In this example, it can be seen that the use of a parametric or a non-parametric test leads to the same conclusion regarding statistical significance (Table 5). As can be observed in Figure 5, the correlation between the *t*-test and chi-square test is good and approximately linear.

## 4. Conclusions

Likert data from an actual survey are neither continuous nor Gaussian in distribution, and numbers per subgroup vary widely. In spite of this, parametric analyses are “robust” [14] as judged from the observation that parametric and non-parametric analyses lead to similar conclusions regarding statistical significance. The explanation for this may lie in the fact that numbers are large and distributions are similar.

Graphical representation in the form of scores provided an easier visual appreciation of differences. The calculation of scores, however, leads to a loss of information as a 4-point Likert scale is transformed into a binary scale. We suggest that this could be “compensated” by determining the difference between scores on the basis of a non-parametric chi-square test on the original ranking data.

Applying parametric analysis of real survey data leads practically in all cases to the same conclusions as those drawn from applying non-parametric analyses. Thus, the advantages of parametric analysis [15], which as seen above is more discriminant, can be exploited in a robust fashion. Several authors have criticized this position and argued on theoretical grounds that parametric analysis of ordinal data such as Likert rankings is inappropriate [4]. Others, after extensive analysis, have reached different conclusions. Thus, Glass et al. [16] concluded that “the flight to non-parametrics was unnecessary principally because researchers asked ‘are normal theory ANOVA assumptions met?’ instead of ‘how important are the inevitable violations of normal theory ANOVA assumptions?’” In this paper, we have attempted to follow the same pragmatic approach. Likewise, Norman [9], after dissecting the argument that parametric analysis cannot be used for ordinal Likert scales, reached the conclusion that “parametric statistics are robust with respect to violations of these assumptions parametric methods can be utilized without concern for ‘getting the wrong answer.’” Finally, Carifio and Perla [17], after considering the arguments, counter-arguments and empirical evidence found “many persistent claims and myths about ‘Likert scales’ to be factually incorrect and untrue.”

In the light of the above, we suggest that, in the case presented here, the use of scores for graphical representation plus chi-square for analysis of Likert data, which (1) facilitates the visual appreciation of the data and (2) avoids the futile “parametric” versus “non-parametric” debate, assured the best mosaic of statistical tests combined with phenomenological analysis.

In our example, sample sizes are large (=/>72) and the question can be asked as to how sample size could affect our conclusions. It is certain that, according to the laws of large numbers, experimental frequencies tend in probability to theoretical probability, but the rapidity of such convergence was not our aim. The problem of sample size was discussed by Boneau [7], who suggested that “samples of sizes of 15 are generally sufficient to undo most of the damage inflicted by violation of assumptions. Only in extreme cases involving distributions differing in skew [authors’ note: as was the case in our example] would it seem that slightly larger sizes are prescribed say, 30, for extreme violations.” It should be noted, however, as discussed by Norman [9], that, “Nowhere is there any evidence that non-parametric tests are more appropriate than parametric tests when sample sizes get smaller.” Curtis et al. argued—on theoretical grounds—that (more or less equal) numbers per group is also an important factor for ensuring robustness of statistical analysis [18]. Again, in our pragmatic approach, sample sizes varying from 72 to 188 did not appear to affect the issue.

Another possible issue concerns homogeneity of variance given that the IP data show some differences in distribution to those of the other three subgroups. This does not seem to be a problem given the similarities between the parametric and non-parametric analyses of CP versus IP. This is in agreement with the work of Boneau [7], on simulated data, who concluded “that for a large number of different situations confronting the researcher, the use of the ordinary t test and its associated table will result in probability statements which are accurate to a high degree, even though the assumptions of homogeneity of variance and normality of the underlying distributions are untenable. This large number of situations has the following general characteristics: (a) the two sample sizes are equal or nearly so (authors’ note: this was not the case in our example); (b) the assumed underlying population distributions are of the same shape or nearly so.”

## Figures and Tables

**Table 1:** Numbers of negative and positive values for “number of values for Rank 4–number of values for Rank 3”, range, means, standard deviations, and Kolmogorov–Smirnov test for normality, in the four subgroups of pharmacists.

Subgroup	CP	HP	IP	OP
Numbers of inverted j distributions	24	25	39	28
Numbers of linear/exponential distributions	26	25	11	22
Mean of values Rank 4–Rank 3	0.2	1.6	−7.7	−1.1
Standard deviation	27	37	15	12
Kolmogorov–Smirnov (KS) normality test				
KS distance	0.085	0.11	0.12	0.12
Passed normality test (alpha = 0.05)?	Yes	Yes	Yes	Yes

**Table 2:** Parametric (top) and non-parametric (bottom) analyses of the significance of the effect of competency using the ranking data for CP (*n* = 183).

**Parametric**					
**1-Way ANOVA**	**Sum of Squares**	**Degrees of Freedom**	**Mean Square**	**F (49, 8045)**	***p*-Value**
Treatment (competencies)	611.2	49	12.47	22.99	*p* < 0.0001
Residual	4365	8045	0.5426		
Total	4976	8094			
**Non-Parametric**					
**Kruskal–Wallis Test**					
*p*-value (for competencies)				<0.0001	
Kruskal–Wallis statistic				720.8	

**Table 3:** Comparison of the significance of the differences amongst rankings for competencies within subgroups obtained by the parametric Bonferroni and the non-parametric Dunn tests (data for CP).

		Dunn	Dunn	
		Significant	Not significant	Total
Bonferroni	Significant	393	76	469
Bonferroni	Not significant	0	756	756
	Total	393	832	1225

**Table 4:** Parametric (upper) and non-parametric (lower) analyses of ranking data for four subgroups of pharmacists. (**a**) Parametric two-way ANOVA and Sidak’s multiple comparisons test for differences amongst subgroups (number of missing values: 14,328). (**b**) Non-parametric Friedman analysis with Dunn’s multiple comparisons test for differences amongst subgroups.

**(a)**													
**ANOVA Table**	**Sum of Squares**	**% of Total Variation**		**Degrees of Freedom**		**Mean Square**		**F**			***p***		
Interaction: competency–subgroup	289	2.1		147		2.0		F (147, 22,872) = 3.6			*p* < 0.0001		
Competency	1032	7.3		49		21		F (49, 22,872) = 38			*p* < 0.0001		
Subgroup	17	0.12		3		5.7		F (3, 22,872) = 10			*p* < 0.0001		
Residual	12,517			22,872		0.55							
**Sidak’s Multiple Comparisons Test, Comparisons with CP Only Are Given**		**Difference of Means**		**95% Confidence Limits of Difference**				***p*-Value Summary**					
CP versus HP		0.0087		−0.019 to 0.036				Not significant					
CP versus IP		0.0630		0.029 to 0.098				*p* < 0.0001					
CP versus OP		0.0520		0.014 to 0.090				*p* < 0.01					
**(b)**													
**Friedman Statistic**							**10.05**						
*p*-value							0.0182						
Number of subgroups							4						
**Dunn’s Multiple Comparisons Test, Comparisons with CP Only Are Given**			**Rank Sum 1**		**Rank Sum 2**		**Sum Difference**		**N1**	**N2**		***p***	
CP versus HP			139.0		139.0		0.0		50	50		*p* > 0.05	
CP versus IP			139.0		106.0		33.00		50	50		*p* > 0.05	
CP versus OP			139.0		116.0		23.00		50	50		*p* > 0.05	

**Table 5:** Comparison of the chi-square test with the parametric *t*-test for the differences in competencies between CP and IP. For both tests, all values are *p* < 0.05.

Competency	*t*-Test	Chi-Square
21	3.49	17.2
22	4.99	22.9
23	5.18	27.9
28	2.93	10.4
29	3.63	13.7
30	3.47	12.1
